# Effects of Metformin on the Regulation of Free Fatty Acids in Insulin Resistance: A Double-Blind, Placebo-Controlled Study

**DOI:** 10.1155/2012/394623

**Published:** 2012-10-11

**Authors:** Manuel Castro Cabezas, Jeroen P. H. van Wijk, Jan Willem F. Elte, Boudewijn Klop

**Affiliations:** ^1^Department of Internal Medicine, Center for Diabetes and Vascular Medicine, Sint Franciscus Gasthuis Rotterdam, P.O. Box 10900, 3004 BA Rotterdam, The Netherlands; ^2^Department of Internal Medicine, Gelderse Vallei Hospital, P.O. Box 9025, 6710 HN Ede, The Netherlands

## Abstract

*Introduction*. Impaired free fatty acid (FFA) metabolism is closely linked to insulin resistance. Our aim was to evaluate plasma FFA changes in insulin resistance in a physiological situation after improvement of insulin sensitivity by metformin. *Methods*. A double-blind, placebo-controlled intervention with metformin was carried out in patients with insulin resistance. Nineteen patients were randomized to receive metformin 850 mg b.i.d. during 6 weeks or placebo. Participants underwent a mental stress test and an oral glucose tolerance test (OGTT) before and after treatment. *Results*. Fasting plasma glucose, FFA, and HOMA-IR tended to decrease after metformin, suggesting improved insulin sensitivity. FFA concentrations during the mental stress test showed a similar pattern after metformin, albeit lower at all time points, in contrast to the placebo group. The decrease in fasting plasma FFAs was positively associated to the decrease in HbA1c (*r* = 0.70; *P* = 0.03) and in fasting glucose (*r* = 0.74; *P* = 0.01). The suppression of plasma FFAs during OGTT did not change by metformin or placebo. *Conclusion*. Metformin in insulin resistance did not lead to improved FFA dynamics despite a trend of improved insulin sensitivity. Metformin most likely decreases plasma FFAs mainly by suppressing fasting FFA concentrations and not by suppression of acute stress-induced lipolysis.

## 1. Introduction

Free fatty acids are generated during hydrolysis of mono-, di-, and triglycerides by a regulatory network of lipid and energy homeostasis [1]. Lipolysis *in vivo* is mediated by different intracellular enzymes in the adipocyte, endothelial lipases, adipocyte cell size, and blood flow in adipose tissue [2–7]. FFA plasma concentrations change within certain physiological limits mainly based on the hormonal balance in each individual [8, 9]. Mental stress is one of the physiological stimuli, besides other physiological factors such as the nutritional state [10], physical activity [11], or ageing [12] leading to higher plasma FFA concentrations [13]. The mechanism whereby mental stress acutely increases FFAs, is stimulation of catecholamine secretion leading to enhanced hydrolysis of intracellular triglycerides in adipose tissue [13, 14]. The counterpart of this activating system is insulin, which leads to an acute inhibition of intracellular lipolysis and therefore, suppression of FFA plasma levels [8, 13, 14]. In situations of insulin resistance, both stimulation and inhibition of intracellular FFA hydrolysis are impaired [15, 16]. It has been proposed that increasing insulin sensitivity with metformin may result in improved FFA metabolism at the level of the adipocyte [17]. Metformin has been proposed to acutely inhibit catecholamine-stimulated lipolysis in insulin resistant subjects [18]. However, it remains unclear if the FFA lowering effect of metformin is based on lowering basal FFA concentrations or by improving FFA dynamics during acute lipolysis. The use of a mental stress test potentially helps to investigate basal FFA concentrations and FFA dynamics due to catecholamine-stimulated lipolysis under physiological conditions. The present investigation was a double-blind placebo controlled study, designed to determine plasma FFA dynamics under mental stress and during high insulin levels before and after improvement of insulin sensitivity with metformin.

## 2. Materials and Methods

### 2.1. Subjects and Design

The study protocol was approved by the Human Investigations Review Committee of the University Medical Centre Utrecht (WOM 00/066). Nineteen insulin resistant subjects were recruited from the Endocrinology out-patient clinic of the University Medical Centre Utrecht and by advertisement. The major inclusion criteria were the presence of the metabolic syndrome according to the NCEP guidelines [19] or type 2 diabetes mellitus regulated by sulfonylurea derivatives. Excluded were patients with liver, renal, or thyroid failure, manifesting cardiac failure, or a daily alcohol intake of 3 units or more, and patients using metformin. 

On the morning of inclusion weight, height, blood pressure, and waist-to-hip ratio were measured and fasting, venous blood samples were drawn for the determination of lipids, apolipoproteins, glucose, FFA, and insulin concentrations.

After a two-week run-in period in which all participants used placebo two times daily, patients were double-blind randomized to metformin 850 mg b.i.d. or placebo. These were kindly donated by Merck-Lipha, Chilly-Mazarin, France. All participants were allowed to continue their medication. The first mental stress test and oral glucose tolerance test (OGTT) were performed during the run-in period in the first week after inclusion. The second series of experiments were done after 6 weeks of treatment with either metformin or placebo. Participants visited our department every two weeks to encourage participation, for evaluation of safety parameters, and for pill counting.

### 2.2. Mental Stress Tests

All subjects underwent a mental-stress test as described [13, 14, 16]. The participants visited the metabolic ward of our laboratory after a 12-hours overnight fast, where an intravenous cannula was placed in a brachial vein. The cannula was kept open by a continuous 0.9% saline infusion. All peripheral blood samples were obtained from the cannula in sodium EDTA (2 mg/mL), placed on ice, and centrifuged immediately for 15 min at 3000 RPM at 4°C. An inhibitor of lipoprotein lipase (tetrahydrolipstatin, Roche, Switzerland) was added to the plasma immediately after centrifugation in order to block *in vitro* lipolysis [20]. The subjects remained supine in a room without disturbing stimuli during the first 60 minutes of the test. The next twenty minutes, the participants were subjected to two types of mental-stress tests consisting of letters and figures as described in detail elsewhere [13, 14, 16]. After the mental stress period, the subjects remained 40 minutes supine. Peripheral blood samples were obtained before the mental stress period (*T* = −60 to 0 minutes) at 10 minutes intervals. During the twenty minutes of mental-stress (*T* = 0 to 20 minutes) blood samples were taken with an interval of 5 minutes and after the stress-period (*T* = 20 to 40 minutes) at 10 minute intervals. The heart rate was also recorded during the test.

### 2.3. Oral Glucose Tolerance Test

Immediately after the mental stress test an OGTT was performed. The subjects ingested a solution of 300 mL containing 75 gram glucose. Blood samples were collected at baseline and at 30 minute intervals up to 120 minutes for determination of FFA, glucose, and insulin concentrations.

### 2.4. Analytical Methods

Plasma samples were stored at –20°C immediately after centrifugation. FFA concentrations were measured in duplicate in plasma samples by enzymatic colorimetric method (Wako Chemicals GmbH, Neuss, Germany). Insulin concentrations were measured by commercial ELISA (Mercodia, Uppsala, Sweden). Plasma glucose was measured by glucose oxidase dry chemistry (Vitros GLU slides) and colorimetry. Cholesterol and triglycerides were determined using a Vitros 250 analyzer (Johnson & Johnson Rochester, NY, USA). Plasma apo B was measured by nephelometry using apo B monoclonal antibodies (Behring Diagnostics NV, OSAN 14/15). For estimation of insulin sensitivity the HOMA-IR (=glucose × insulin/22.5) was calculated [21].

### 2.5. Statistics

All values are expressed as mean ± standard error of the mean (SEM). Differences between the groups were tested by ANOVA. Normality was tested with the Kolmogorov-Smirnov-test and if non-normality occurred, as in the case of plasma TG, plasma insulin and HOMA-IR, calculations were performed after logarithmic transformation. For statistical analysis of changes in FFA concentrations repeated measures ANOVA was used, with LSD test as post-hoc analysis test. For estimation of total amount of FFA released by mental stress, the area under the FFA curve (AUC) and the area under the FFA curve corrected for the FFA concentration at *T* = 0 (dAUC) were calculated for the stress period. The AUC for glucose during the OGTT was calculated. Graphpad Prism 5.0 (GraphPad Software Inc., La Jolla, CA, USA) was used to calculate both the AUC and dAUC. Pearson's correlation coefficients (*r*) were calculated to investigate associations between FFA changes and other variables. Statistical significance was reached when *P* < 0.05 (two-tailed). Statistical analyses were performed using PASW Statistics 18.0 (IBM, New York, NY, USA).

## 3. Results

### 3.1. General Characteristics (Table 1)

Nineteen patients with insulin resistance completed the study. Five patients with type 2 diabetes mellitus were included in the metformin group and two in the placebo group. The general characteristics of the participants are given in Table 1. There were no significant differences between the groups. After 6 weeks of treatment with metformin, fasting free fatty acids, plasma glucose, HbA1c, HOMA-IR, and total cholesterol tended to decrease without reaching statistical significance. Metformin tended to decrease fasting plasma FFA from 0.61 ± 0.08 to 0.44 ± 0.06 mmol/L (*P* = 0.06) in contrast to placebo (from 0.58 ± 0.22 to 0.55 ± 0.07 mmol/L), suggesting improved insulin sensitivity by metformin. In the placebo group, all fasting plasma variables remained unchanged.

### 3.2. Plasma FFA Concentrations during the Mental Stress Test (Figure 1)

Heart rate increased by 9.8% in the metformin group (*P* = 0.005) and by 8.8% in the placebo group (*P* = 0.07) during the mental stress test with the maximum increase at 5 to 10 minutes after the start of the mental stress. During the first 60 minutes before the mental stress, FFA plasma levels did not change significantly. During mental stress, plasma FFAs increased significantly in both groups resulting in similar increments after each intervention. There were no significant effects by metformin on mental stress-induced FFA changes when compared to placebo. During the 20 minutes of mental-stress, FFA increased significantly in the metformin group by 30.9 ± 2.1% before and 29.8 ± 1.6% after treatment and in the placebo group by 31.7 ± 3.1% and 34.1 ± 3.3%, respectively. The incremental area under the FFA curve showed no significant difference between the groups (1.77 ± 0.49 mmol·min/L before and 1.39 ± 0.29 mmol·min/L after metformin versus 1.39 ± 0.38 mmol·min/L before and 1.60 ± 0.41 mmol·min/L after placebo). The variables best associated with the decrease of fasting plasma FFA after metformin were the decrease in plasma glucose (*r* = 0.74; *P* = 0.01) and in HbA1c (*r* = 0.70; *P* = 0.03).

### 3.3. Glucose, Insulin and FFA Concentrations during OGTT (Figure 2)

Glucose plasma levels increased during OGTT until approximately 90 minutes, and showed subsequently a gradual decrease. The AUC for glucose during the OGTT was slightly decreased after treatment with either metformin or placebo without reaching statistically significant results (1427.2 ± 497.2 mE·min/L before and 1215.0 ± 318.7 mE·min/L after metformin versus 1230.9 ± 491.1 mE·min/L before and 1132.0 ± 432.9 mE·min/L after placebo). The postprandial insulin peak was already observed 30 minutes after the start of the OGTT after treatment with metformin, whereas the postprandial insulin peak in the placebo group was at 90 minutes. During OGTT, plasma FFA concentrations decreased in both groups similarly. In the metformin group FFAs were suppressed at 30 minutes by 12.6 ± 2.8% before versus 23.6 ± 3.4% after treatment while in the placebo group 20.4 ± 2.0% before versus 31.6 ± 2.3% after treatment.

## 4. Discussion

Disturbances of FFA metabolism are of major importance in the pathogenesis of insulin resistance as seen in type 2 diabetes mellitus [22–24]. The molecular mechanisms have not been completely clarified [25]. The glucose lowering drug metformin reduces plasma FFA concentrations in patients with type 2 diabetes mellitus [26–29] and improves insulin sensitivity [30, 31]. The question so far was whether this was an adipose tissue-dependent effect or not. Our data are partly in line with earlier reports [26, 27], since fasting FFA were decreased by metformin, albeit not statistically significant, most likely due to the small number of subjects included in our study. This resulted in lower FFA levels throughout the mental stress test when compared to the untreated situation, without affecting the pattern of the curve. The plasma FFA curves in the placebo group were virtually unchanged. Therefore, it seems that metformin decreases plasma FFA mainly by suppressing the fasting levels and not by improving FFA dynamics by acute stress-mediated lipolysis. Most likely the major effects of metformin may be on glucose metabolism at the level of the liver and muscle as suggested by others [32]. Metformin suppresses the hepatic glucose output concomitant to a reduction in intracellular ATP content [32]. In contrast, it has been suggested that part of the insulin sensitizing effect of metformin may be caused by a decrease of plasma FFA [17]. In our study we did not find evidence for *in vivo* suppression of lipolysis by metformin during the mental stress tests. The exact mechanism whereby fasting FFA are decreased by metformin may not fully depend on reduced adipocyte lipolysis *in vivo*. 

Reduction of hepatic glucose output will result in a more efficient “glucose-fatty acid cycle”, which will be accompanied by improvement of FFA handling [25, 33, 34]. This may explain the lower fasting FFA levels by metformin in our study without any effect on the dynamics of acute release (during the mental stress test) and suppression (OGTT). The observed associations between the reduction of fasting plasma FFA and the reductions in glucose and HbA1c support this concept. 

In contrast to our results, a study using microdialysis showed that acute treatment with the same dosage of metformin had an inhibitory effect on catecholamine-mediated lipolysis of abdominal subcutaneous adipose tissue [18]. However, both studies used different methods to establish adipocyte lipolysis. We induced a catecholamine response during the mental stress tests, which is a more physiological stimulus and may therefore not be able to detect small differences. We have not measured catecholamine levels during the mental stress test, but we did observe an increase in heart rate during the test suggesting increased sympathetic activity. An increased catecholamine response induced by an identical mental stress has been described before by our group [13]. Although we did not quantitate directly adipose tissue FFA efflux, the plasma changes in this setting mainly reflect FFA metabolism in the adipose tissue rather than intravascular lipolysis because plasma triglycerides do not change [13, 14, 16]. Another limitation of our study is the relatively small number of subjects included, which could have been responsible for the lack of strength for some comparisons between both groups. Apparently, the placebo group was less insulin resistant than the metformin group. However, both groups showed similar characteristics of insulin resistance. The trend towards an improvement in fasting free fatty acids, plasma glucose, HbA1c, and HOMA-IR was only observed in the metformin group. We would have expected that these results would translate into improved FFA kinetics in this group, as observed by others [18].

In conclusion, fasting FFAs were lower after metformin, but the dynamics of FFAs by acute physiologic stimuli did not change. Therefore, we propose that metformin decreases plasma FFAs *in vivo* mainly by suppressing baseline FFA concentrations and not by suppression of acute stress-mediated lipolysis.

## Figures and Tables

**Figure 1:**
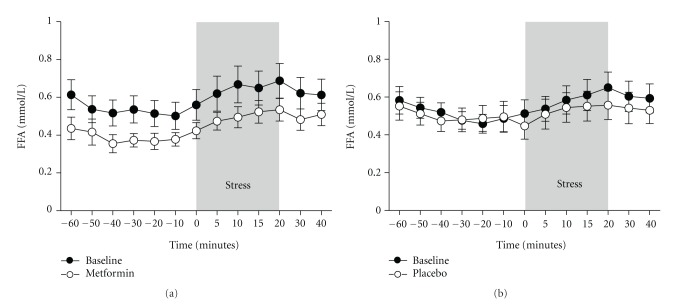
Mean changes in free fatty acid (FFA) concentrations during the mental-stress test (MST) in insulin resistant patients at baseline and after six weeks of treatment with metformin (*N* = 10) (a) or placebo (*N* = 9) (b). Data represent the mean ± SEM.

**Figure 2:**
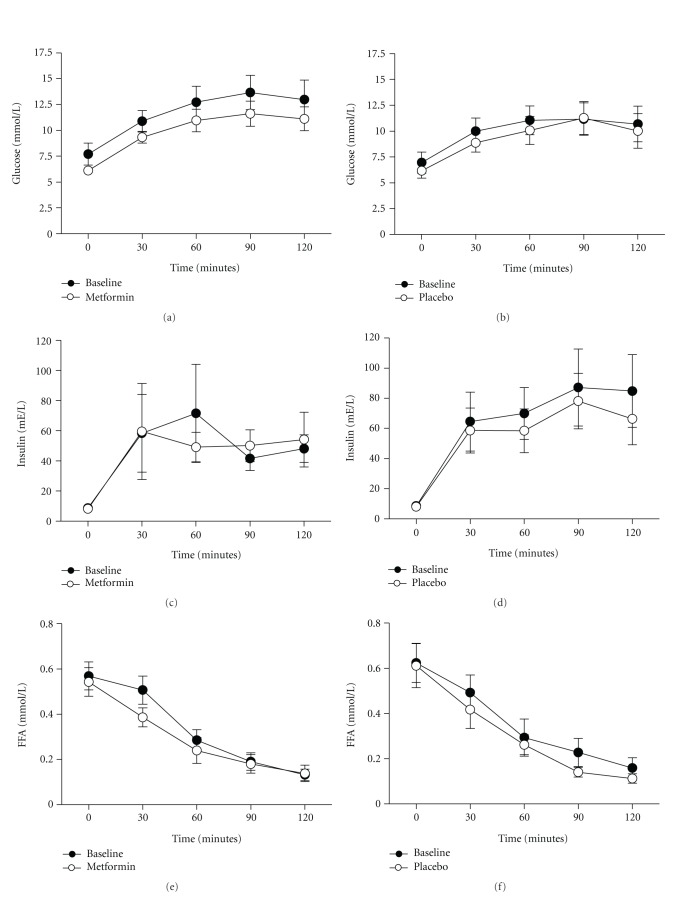
Mean change in glucose, insulin, and free fatty acid (FFA) concentrations during an oral glucose tolerance test (OGTT) in insulin resistant patients at baseline and six weeks after treatment with either metformin (*N* = 10) (a, c, and e) or placebo (*N* = 9) (b, d, and f). Data represent the mean ± SEM.

**Table 1:** Characteristics of patients included in the metformin and the placebo groups at baseline and six weeks after treatment. Data are given as mean (SEM) unless stated otherwise.

	Metformin (*N* = 10)		Placebo (*N* = 9)	
	Untreated	Treated	Untreated	Treated
Gender (M/F)	5/5	—	5/4	—
Age (years)	49 (3)	—	53 (3)	—
BMI (kg/m^2^)	32.0 (1.4)	32.0 (1.4)	30.4 (1.4)	30.3 (1.4)
WHR	1.03 (0.02)	1.02 (0.02)	0.96 (0.03)	0.95 (0.03)
Systolic BP (mmHg)	137 (4)	132 (4)	143 (7)	139 (4)
Diastolic BP (mmHg)	88 (3)	87 (3)	90 (4)	86 (5)
Fasting FFA (mmol/L)	0.61 (0.08)	0.44 (0.06)^#^	0.58 (0.07)	0.55 (0.07)
Total cholesterol (mmol/L)	5.4 (0.5)	4.8 (0.4)^$^	5.8 (0.5)	5.4 (0.4)
HDL cholesterol (mmol/L)	1.12 (0.11)	1.14 (0.07)	1.22 (0.17)	1.21 (0.16)
Plasma triglycerides (mmol/L)	1.91 (0.30)	1.69 (0.30)	2.04 (0.36)	2.08 (0.36)
Apo A1 (mg/L)	1.27 (0.09)	1.17 (0.04)	1.36 (0.10)	1.22 (0.10)
Apo B (mg/L)	1.05 (0.11)	0.91 (0.09)	1.05 (0.10)	1.04 (0.10)
Fasting plasma insulin (mE/L)	19.2 (5.2)	18.2 (6.7)	14.4 (3.2)	14.3 (2.5)
Fasting plasma glucose (mmol/L)	8.4 (1.0)	6.8 (0.5)*	7.1 (1.1)	7.1 (0.7)
HOMA-IR	6.4 (2.6)	5.4 (2.4)*	4.4 (1.0)	4.4 (0.8)
GlyHb (%)	6.8 (0.4)	6.4 (0.3)^&^	6.0 (0.4)	6.3 (0.3)

^#^ *P* = 0.06; *P = 0.07; ^&^ *P* = 0.08; ^$^ *P* = 0.05.

Abbreviations: BMI: body mass index; WHR: waist-to-hip ratio; BP: blood pressure; FFA: free fatty acid; Apo: apolipoprotein; HOMA-IR: homeostasis model assessment of insulin resistance.
